# Extended-release amphetamine (Dyanavel XR) is associated with reduced immediate-release supplementation in adults with ADHD, regardless of baseline patient variables: a retrospective cohort analysis of medical treatment records

**DOI:** 10.1186/s12888-024-06446-z

**Published:** 2025-01-03

**Authors:** Joel L. Young, Richard N. Powell, Anna Powell, Lisa L. M. Welling, Lauren Granata, Jaime Saal, Margot Nash

**Affiliations:** 1 Rochester Center for Behavioral Medicine, Rochester Hills, USA; 2 MedaData, LLC, Rochester Hills, USA; 3 Department of Psychology, Oakland University, Rochester, USA; 4 School of Medicine, Wayne State University, Detroit, USA

**Keywords:** ADHD, Adult treatment, Regression analysis, Extended-release, Immediate-release, Amphetamine, Methylphenidate

## Abstract

**Background:**

Adults with ADHD benefit from treatment with extended-release (ER) formulations that provide symptom control for the entire day. Some patients are advised to supplement their extended-release medication with an immediate-release (IR) medication later in the day if they need to prolong its effects. Given that several FDA-approved ER formulations are available and many individual patient variables may affect efficacy, the purpose of this study was to identify reliable predictors of the tendency for patients to supplement their daily ER medication with an IR medication.

**Methods:**

This retrospective study analyzed data from medical treatment records of adults with ADHD who received at least one ER psychostimulant (amphetamine or methylphenidate preparations) for at least six months between November 2022 and June 2024 (*N* = 417). Data from their intake evaluations, pre-visit measures of depression, anxiety, and ADHD via validated self-report scales, and post-visit clinician evaluations were compiled from their electronic medical records and the Qualtrics API. The association between Dyanavel XR, IR supplementation, and patient variables were investigated by backward stepwise linear regressions modeled using the variable groupings: (1) side effects reported at baseline, (2) side effects reported after 90 days, and (3) change in depression, anxiety, and ADHD symptoms from baseline to 90 days using assessment scale scores.

**Results:**

Compared to the other amphetamine and methylphenidate ER medications, only Dyanavel XR resulted in lower IR supplementation at 90 days. This relationship held when controlling for baseline IR use. Regardless of whether patients supplemented with an IR, they demonstrated improved ADHD symptoms as measured by the ADHD Symptom and Side Effect Tracking (ASSET) scale after 90 days (*d* = 0.68 in patients with IR, *d* = 0.39 in patients without IR). Dyanavel XR was significantly associated with reduced IR supplementation at 90 days compared to the pooled group of patients taking other ER medications (χ^2^ = 4.320, Nagelkerke *R*^2^ = 0.039, *p* = .038). The CGI-I score at baseline was also significantly associated with supplementation at 90 days (*r* = .14, *p* = .010). No other baseline variable was independently associated with IR supplementation. The combination of being on Dyanavel XR, having negative changes in ADHD, and positive changes in anxiety symptom presentation from the baseline to the 90-day visit predicted reduced IR supplementation (ASSET change: *t* = 2.377, *p* = .018; GAD-2 change: *t* = -2.543, *p* = .011; Dyanavel XR: *t* = -2.112, *p* = .035).

**Conclusion:**

These analyses support Dyanavel XR as a monotherapy for the daily management of ADHD in adults compared with other ER medications. Considering its tendency to reduce IR supplementation, Dyanavel XR may simplify treatment regimens and improve outcomes.

**Clinical trial number:**

Not applicable.

## Background

ADHD is most commonly treated with stimulant and non-stimulant medications, which have been shown to be clinically effective [1]. Extended-release (ER) stimulants provide the benefits of long-acting symptom control leading to greater treatment satisfaction compared to immediate-release (IR) stimulants [2]. These long-acting medications reduce the need for repeated doses, thereby improving adherence and treatment response [3–5]. They also have a lower risk of misuse compared to short-acting IR stimulants [6]. However, IR stimulants are still used to supplement once-daily medications when symptoms are not sufficiently controlled [6, 7]. Prescribers may advise using IR formulations to prolong and boost the therapeutic effects of an ER medication or to curb unwanted effects if the ER dose wears off [6, 7]. 

Formal treatment guidelines specifically for adults with ADHD have not yet been developed in the U.S [8]. With approximately 30 different FDA-approved stimulants available for providers to choose from, first-line treatment decisions are often the result of trial and error [8–10]. Justifying an initial choice of prescription is further complicated by the variability in treatment responses between patients [11]. Although stimulants are the most effective intervention for ADHD [1], some patients do not respond well to common frontline medications [12]. A number of clinical factors contribute to heterogeneous treatment effects. Individuals who are older (amongst children), have milder symptoms, and have comorbid anxiety are the least likely to respond well to stimulants [13]. Worse initial symptoms, including inattentiveness and disinhibition, are weak predictors of favorable responses [13]. Treatment non-adherence is related to worse outcomes. Patients are less likely to adhere to treatment if they are younger (< 25 years old), have less than a secondary level of education, lack of family history of ADHD, have lower baseline symptom severity, and perceive lower medication efficacy [14, 15]. 

The rise of precision medicine in psychiatry has underscored the need to identify reliable predictors of treatment response, particularly the tendency to supplement daily medication with IR formulations [12]. Previous studies have shown that ER medications are effective on the group level, but few have sought to find whether individual variability can be attributed to baseline patient characteristics [16, 17]. Variable clinical outcomes likely arise due to complex interactions between patient factors, including baseline psychological profiles and demographics [12]. Demonstrating reduced IR supplementation with a particular medication, regardless of underlying individual variables, would provide a basis on which to make first-line treatment decisions. If ER efficacy is significantly altered depending on a specific patient variable, it would help inform individualized treatment plans.

Dyanavel XR is an ER amphetamine with a targeted pharmacokinetic profile enabling rapid onset of action with continuous release that prolongs its active duration to allow once-daily dosing [18]. Dyanavel XR leverages its unique technology to optimize the balance between fast onset of effect and maintenance of efficacy throughout the day [19, 20]. In adults, Dyanavel XR improves symptoms and has a safety profile comparable with other approved stimulants [18]. In a large national sample representing 60% of all insurance claims in the U.S., Dyanavel XR was shown to be more frequently used as monotherapy compared to other ER medications [21]. 

The abundance of individual factors with the potential to confound the overall effect of Dyanavel XR on monotherapy rates warrants investigation to determine whether any patient-level variable can explain the effect of Dyanavel XR on rates of IR supplementation. The purpose of the current study was to determine whether Dyanavel XR’s tendency to reduce IR supplementation could be explained by any other individual patient variable. Through a series of predictive analyses using retrospective data from 417 adult patients with ADHD from the Rochester Center for Behavioral Medicine (RCBM), the results aim to provide insights into potential predictive variables for ADHD treatment response.

## Methods

### Study design

This study is a retrospective cohort analysis of medical treatment records obtained between November 2022 and June 2024.

### Participants

To be included, participants must have had a diagnosis of ADHD, have received treatment for ADHD, be at least 18 years of age or older at the start of treatment, received treatment of at least one extended release (ER) psychostimulant (amphetamine or methylphenidate preparations) for at least six months, and provided consent for secondary research use of their medical treatment data. Participants with a potentially confounding comorbid psychiatric condition, including bipolar spectrum disorders, alcohol and substance use disorders, or an initial Patient Health Questionnaire-9 (PHQ-9) [22] score greater than or equal to 14, were excluded. Participants with a potentially confounding comorbid medical condition, including thyroid conditions, cancer or chemotherapy treatment, sleep disorders, or migraines, were also excluded from the study.

A stratified sampling strategy was used, grouping patients by ER medication to target a total of 150 patients for each of the following ER formulations: Dyanavel XR, ER amphetamine (equal numbers of Adderall XR and lisdexamfetamine [Vyvanse]), and ER methylphenidate (equal numbers of Focalin, Concerta, and generic methylphenidate ER). The target sample size was calculated based on a power analysis for structural equation modeling to meet a power of 85% when the root mean square of error of approximation is 0.09 and approximated degrees of freedom is 20 [23]. Patient records were selected randomly for each stratum. After reviewing patient records and excluding those who did not meet the study criteria, there were 143 Dyanavel XR, 131 ER amphetamine (65 Adderall XR and 66 lisdexamfetamine), and 143 methylphenidate (51 Focalin, 53 Concerta, and 39 generic methylphenidate ER) patient records meeting the inclusion criteria. If patients were missing any assessments, their data were included in analyses where possible, but values were excluded if missing.

### Procedure

Electronic medical records were obtained from Rochester Center for Behavioral Medicine (RCBM), a large outpatient psychiatric practice, which maintains a HIPAA-compliant Qualtrics platform for securely administering patient-facing forms, questionnaires, and psychometrics. The study data elements were retrieved via direct queries to the electronic medical records system’s server and queries to the practice’s Qualtrics system’s electronic storage. Pharmacy data is sent and received through the SureScripts system.

Data were de-identified by compiling the elements onto a single datasheet as they are retrieved, where one row corresponded with one patient. No columns on this data sheet contained any of the 18 identifiers described in the HIPAA privacy rule’s safe harbor provision, ensuring that, to the furthest extent possible after completion of the data sheet, no reidentification of a patient would be possible. The study staff responsible for compiling the datasheet did not retain any records of patient identifiers that may have been consulted in the construction of the study datasheet. No re-identification of a patient was attempted or permitted by the study researchers.

Participants were referred to RCBM prior to treatment by various mental health and medical professionals and completed intake forms prior to their intake appointment. Next, they completed a pre-visit survey prior to each visit through the online survey distribution software, Qualtrics. The pre-visit survey is hosted by RCBM’s Qualtrics platform and is connected with RCBM’s electronic charting program through the Qualtrics API. This API connection allows for a patient’s individual responses to be automatically filed in their chart for clinician review. Their responses are paired with information from the electronic medical record so the clinician can review the patient-reported information in tandem with their medical history and clinician-reported symptom severity ratings from visit to visit. The API also allows the researchers to extract the necessary variables for the current study’s analyses without accessing the electronic medical record system. This analysis compiled data from the pre-visit surveys at baseline and after 90 days of ER treatment. The follow-up time point of 90 days was selected because, in practice, most patients would have had their first follow-up appointment with their prescribing clinician within 90 days [24]. 

### Assessment instruments

#### Patient history and information collected prior to initial visit

Prior to their initial visit at RCBM, patients completed intake forms reporting their demographic information, including age, gender, natal sex, employment status, education completed, marital status, and ethnicity. They also self-reported their prescription history prior to RCBM. Clinician-reported information was queried from the electronic medical record database, which included the patients’ prior diagnoses, current diagnoses, and current prescriptions.

#### Clinician-reported information

Clinicians completed the Clinical Global Impression (CGI) for patients at each visit [25]. The CGI was developed by the National Institute of Mental Health in collaborative pharmacology trials of schizophrenia to assess illness improvement. Since its origins, it has become a routine measure in psychiatric settings. The scale has three items: Severity of Illness (CGI-S), Global Improvement (CGI-I), and Efficacy Index. CGI-S is a single item rating on a seven-point scale from 1 (“normal”) to 7 (“extremely ill”) asking the clinician to rate the patient’s severity of illness based on their experience with individuals of the same clinical population. The CGI-I is also a single item rating on a seven-point scale from 1 (“very much improved”) to 7 (“very much worse”). The Efficacy Index is a rating of the effect of the therapeutic intervention from 1 (“none”) to 4 (“outweigh therapeutic effect”) [25]. The CGI scale has established utility in the rating of schizophrenia, panic disorder, depression, obsessive compulsive disorder, and social anxiety disorder [26, 27]. It has good concurrent validity and sensitivity to change in patients with panic disorder and depression [28] and performs similarly to other standard outcome measures, including the Health of the Nation Outcome Scales and the Brief Psychiatric Rating Scale [29]. The change between CGI-S score at admission and discharge is highly correlated with the CGI-I at discharge, showing its reliability to interpret changes in disorders [26]. 

### Patient-reported information

Patients completed an online survey prior to every clinic visit. The pre-visit survey form includes three psychometric tools: the Patient Health Questionnaire-9 (PHQ-9) [22], the Generalized Anxiety Disorder-2 (GAD-2) [30], and the ADHD Symptom and Side Effect Tracking (ASSET) scale [31, 32]. To extract patient data for this analysis, researchers performed a query of patient data from the Qualtrics API. The query included the list of medications being actively managed by RCBM at the time of each visit, the type of treatment provider that the patient saw for the follow up visit and/or their prescribing clinician, and the diagnoses and/or clinical problems the clinician designated as the targets of treatment. Data from pre-visit surveys were also used to calculate the tendency of prescribers to pair an IR stimulant with an ER stimulant, as reported by the patients’ indication of their prescribing clinician and current medications.

#### PHQ-9

The Patient Health Questionnaire (PHQ)-9 is a nine-item self-administered screening tool for depression [22]. Responses are rated on a Likert scale from 0 (“not at all”) to 3 (“nearly every day”) indicating increasing severity of symptoms with a maximum score of 27. Across 14 validation studies conducted in primary care, medical outpatients, and specialty services, the PHQ-9 has high sensitivity (0.80 [95% CI: 0.71–0.87]) and specificity (0.92 [95% CI: 0.88–0.95]) for major depression when scores are greater than or equal to 10 [33]. 

#### GAD-2

The Generalized Anxiety Disorder (GAD) scale-2 is a 2-item shortened version of the GAD-7, a seven-item, Likert scale for identifying GAD, with items rated from 0 (“not at all”) to 3 (“nearly every day”) [30, 34]. The GAD-7 has been validated in large samples in primary care, with internal consistency, good test-retest reliability, and high sensitivity (89%) and specificity (82%) for GAD. The GAD-2 was developed as a truncated version of the full questionnaire, only presenting the two questions of the GAD-7 representing the core symptoms of anxiety. With a cutoff score of greater than or equal to three indicating GAD, the GAD-2 scale maintains high sensitivity (86%) and specificity (83%) [35]. 

#### ASSET

The ADHD Symptom and Side Effect Tracking Scale (ASSET) is a ten item self-report measure for ADHD symptom severity with a companion list of assorted side effects clinicians are advised to track throughout psychopharmaceutical treatment for ADHD. The scale asks the participant to rate the level of the impact on daily life functioning they may have experienced due to problems with the sign or symptom of ADHD referenced by the item (anchors: 1 = no problem present, 6 = severe impact). The ten items are split into two subscales. The Inattentive Subscale includes the items “attention span,” “forgetfulness,” “follow-through,” “trouble organizing tasks and activities,” “misplacing daily items,” and “productivity.” The Hyperactivity and Impulsivity Subscale includes the items “fidgetiness,” “trouble waiting turn/general impatience,” “anxiety,” and “mood”. The scoring of the baseline scale is a factor score calculated as a weighted sum of the ten severity items. A cut score of greater than or equal to 4.40 achieves high sensitivity (80%) and specificity (80%) [32]. A factor score change of 0.75 indicates reliable change [31]. The list of side effects included insomnia, generalized pain, fatigue, dry mouth, poor appetite, food binges, tics, anger, suspiciousness, restless legs, end of dose crash, return of symptoms as dose wears off, and unwanted changes in weight, and were rated on a Likert scale (1 = never, 5 = always).

### Statistical analysis

Baseline patient characteristics from the screening battery and pre-visit survey at their initial and 90-day visits, or the visit closest to 90 days since the start of their ER treatment, are reported. Descriptive results are stratified by whether the patient supplemented their treatment with an IR stimulant at 90 days. For continuous variables, mean, standard deviation, minimum, and maximum values were calculated, and frequency counts and percentages were calculated for categorical variables.

The relationship between Dyanavel XR stimulant use and the tendency to supplement with IR stimulants was assessed by a crosstabulation with Z-tests for independent proportions. This analysis excluded patients who had used IR medications at baseline. Because Dyanavel XR was uniquely associated with a reduction in IR use at 90 days, the other medications (Adderall XR, Vyvanse, Focalin, and Methylphenidate ER) were collapsed into a single group, and Dyanavel XR use was coded into a binomial variable (Dyanavel XR = 1, other ER medication = 0). To account for IR stimulant use at baseline, an ANCOVA was conducted using Dyanavel XR as the predictor variable and IR at baseline as the covariate.

Independent relationships between each patient variable and IR supplementation rate were assessed using point-biserial Pearson correlations for continuous variables and binomial regressions for categorical variables.

To determine if any factors related to the patient, treatment, side effects, and treatment responses mediated this relationship between Dyanavel XR use and IR use at 90 days, backward stepwise linear regressions were modeled using variable groupings determined a priori in alignment with hypothesized predictive variables, and with IR use at 90 days as the outcome variable. The predictor variable groupings were: (1) side effects reported at baseline, (2) side effects reported after 90 days, and (3) change in symptoms from baseline to 90 days using assessment scale scores (ASSET, CGI-S, CGI-I, GAD-2, and PHQ-9).

All variables in the group were included in the initial regression analysis. At each step, the variable with the lowest level of significance was removed, and the regression was performed again using the remaining variables. This process was repeated until all variables satisfied the significance condition (*p* < .05). The analyses met the assumptions of linearity, independence, and normality of residuals.

## Results

### Participant demographics and baseline characteristics

The total sample included 417 patients (age: M = 36.0 years, SD = 13.5, range = 18–81). Most patients were female (*n* = 280, 67.3%) and white (*n* = 399, 95.9%). Employment status, marital status, and education completed were also collected when possible (Table 1).

**Table 1:** Baseline demographics

	Patients (*N* = 416)
Age in years, mean (SD)	36.04 (13.47)
Gender, n (%)	
Male	135 (32.5%)
Female	280 (67.3%)
Non-binary	1 (0.2%)
Race, n (%)	
Black	5 (1.2%)
Asian	1 (0.2%)
White	399 (95.9%)
Other	6 (1.6%)
Employment Status, n (%)	
College Student	8 (1.9%)
Employed Full Time	125 (30%)
Employed Part Time	14 (3.3%)
Retired	2 (0.5%)
Disabled	2 (0.4%)
Student (other)	21 (5.0%)
Unemployed	10 (2.4%)
No Data	234 (56.3%)
Marital Status, n (%)	
Divorced	9 (2.2%)
Engaged	5 (1.2%)
Married	170 (40.9%)
Partnered	2 (0.5%)
Separated	2 (0.5%)
Single	183 (44.0%)
Widowed	2 (0.5%)
Missing	43 (10.4%)
Education Completed, n (%)	
High school	13 (3.1%)
Undergraduate	104 (24.7%)
Graduate	21 (4.0%)
Middle school	1 (0.2%)
Some college	27 (12.7%)
Missing	255 (60.6%)

### Effects of Dyanavel XR on IR supplementation at 90 days compared to other ER medications

A linear regression was performed to determine the relationship between IR at baseline and the addition of IR supplementation at 90 days. Overall, IR use at baseline was a significant predictor of IR supplementation at 90 days (*R*^2^ = 0.552).

Due to the overall effect of baseline IR, an exploratory analysis was conducted excluding patients who had used IR medications at baseline to determine if Dyanavel XR had a unique effect on the need to supplement with IR medication compared to other ER medications. Results of a cross-tabulation with Z-tests for independent proportions within each ER medication indicated that few patients (*n* = 23) on any ER medication supplemented with an IR at 90 days. Dyanavel XR was the only ER medication that significantly reduced IR supplementation at 90 days (no IR added: *n* = 140; IR added:, *n* = 3; *p* < .05).

Because Dyanavel XR was uniquely associated with a reduction in IR use at 90 days, the other medications (Adderall XR, Vyvanse, Focalin, and Methylphenidate ER) were collapsed into a single group, and Dyanavel XR use was coded into a binomial variable (Dyanavel XR = 1, other ER medication = 0). To account for IR use at baseline, an ANCOVA was conducted using Dyanavel XR as the predictor variable and IR at baseline as the covariate. As expected, the use of IR medication at baseline was associated with the addition of IR at 90 days of treatment (*F*_1,413_ = 5.86, *p* = .016). Controlling for IR use at baseline, there was a significant effect of Dyanavel on IR at 90 days (*F*_1,413_ = 4.67, *p* = .031), with Dyanavel XR being associated with reduced IR use.

### Predictive variables impacting dyanavel XR’s effect on IR supplementation at 90 days

Of the patients who added an IR stimulant at 90 days, their prescribers had an average IR-prescribing tendency of 22.6%, and of patients who did not add an IR at 90 days, their prescribers had an average IR-prescribing rate of 27.3%. At baseline and at 90 days, patients took ASSET, GAD-2, and PHQ-9 tests, and their clinicians completed CGI-S and CGI-I scales (for descriptive statistics of assessment outcomes, see Table 2). Regardless of whether patients added an IR medication at 90 days, ASSET scores improved over the 90-day time period, but the effect was stronger in patients who supplemented with an IR medication (Table 3).

**Table 2:** Descriptive statistics: patients with and without IR supplementation at 90 days^a^

Variable	*n*	Min	Max	Mean	SD
**Patients with IR supplementation at 90 days**					
Time in Treatment Prior to ER (Days)	23	0.08	4761.07	746.11	1109.78
Baseline					
ASSET	23	0.98	5.44	3.76	1.21
PHQ-9	23	0	24	6.43	5.88
GAD-2	23	0.00	6.0	1.57	1.59
CGI-S	21	2	5	3.67	0.66
CGI-I	20	1	4	2.10	0.79
90 days					
ASSET	23	0.97	5.02	3.00	0.98
PHQ-9	23	0	23	5.61	5.37
GAD-2	23	0.00	6.00	1.91	2.11
CGI-S	21	3	4	3.57	0.51
CGI-I	20	1	4	2.15	0.67
Valid N (listwise)	17				
**Patients without IR supplementation at 90 days**					
Time in Treatment Prior to ER (Days)	393	0.021435	4745.88	989.10	1164.93
Baseline					
ASSET	385	0.00	5.98	3.50	1.19
PHQ-9	392	0	21	7.08	4.68
GAD-2	393	0.00	6.00	1.93	1.55
CGI-S	371	2	6	3.83	0.76
CGI-I	360	1	6	2.81	1.13
90 days					
ASSET	385	0.00	5.88	3.12	1.08
PHQ-9	392	0	24	5.65	4.36
GAD-2	393	0.00	6.00	1.68	1.47
CGI-S	371	1	6	3.71	0.82
CGI-I	360	1	5	2.57	0.97
Valid N (listwise)	313				

ASSET, ADHD Symptom and Side Effect Tracking; CGI-S, Clinical Global Impressions-Severity; CGI-I, Clinical Global Impressions-Improvement; ER, extended release; GAD-2, Generalized Anxiety Disorder 2-Item; PHQ-9, Patient Health Questionnaire-9

^a^ The visit falling closest to 90 days after ER stimulant was prescribed

**Table 3:** Magnitude of the change from baseline in psychometric assessments in patients with and without IR supplementation at 90 days^a^

		Change from baseline				
	*n*	Min	Max	Mean	SD	Cohen’s d^b^
**Patients with IR supplementation at 90 days**						
**ASSET**	**23**	**-1.68**	**2.91**	**0.7570**	**1.11**	**0.68**
PHQ-9	23	-10.00	18.00	0.8261	5.04	0.16
GAD-2	23	-6.00	1.00	− 0.3478	1.47	0.24
CGI-S	21	-2.00	1.00	0.0952	0.62	0.15
CGI-I	20	-1.00	1.00	− 0.0500	0.51	0.10
**Patients without IR supplementation at 90 days**						
**ASSET**	**385**	**-2.60**	**4.12**	**0.3858**	**0.98**	**0.39**
PHQ-9	392	-14.00	15.00	1.4311	4.11	0.34
GAD-2	393	-6.00	5.00	0.2468	1.48	0.17
CGI-S	371	-2.00	3.00	0.1186	0.65	0.18
CGI-I	360	-3.00	4.00	0.2361	1.07	0.22

ASSET, ADHD Symptom and Side Effect Tracking; CGI-S, Clinical Global Impressions-Severity; CGI-I, Clinical Global Impressions-Improvement; ER, extended release; GAD-2, Generalized Anxiety Disorder 2-Item; PHQ-9, Patient Health Questionnaire-9

^a^ The visit falling closest to 90 days after ER stimulant was prescribed

^b^ Indicating the magnitude of the change from baseline (small: Cohen’s d = 0.2, medium: Cohen’s* d* = 0.5, large: Cohen’s *d * ≥ 0.8)

The binary variable indicating whether the patient was prescribed Dyanavel XR or another ER was significantly associated with IR supplementation at 90 days (χ^2^ = 4.320, Nagelkerke *R*^2^ = 0.039, *p* = .038). No other continuous (Table 4) or categorical (Table 5) variable was associated with IR supplementation.

**Table 4:** Point-biserial Pearson correlations demonstrating the relationship between each continuous variable and the addition of IR medication at 90 days. Treatment responses representing a change in score between baseline and 90 days were Z-transformed prior to analysis

Variable Group	Variable	Description	Pearson correlation coefficient (*r*)	*R* ^2^	*p*
Demographics	Age	Age in Years	0.02	0.00032	0.716
ER Prescription Decision	Tendency to Prescribe ER	Integer Value: % of patients with an ER prescription	− 0.10	0.0090	0.07
	Time in Treatment Prior to ER	Integer Value	− 0.05	0.0023	0.33
Assessment scores at baseline	ASSET	ADHD symptoms in terms of daily life functioning impact at the time of visit	0.050	0.0025	0.31
	PHQ-9	Severity of depressive symptoms in terms of frequency at the time of visit	− 0.03	0.00096	0.53
	GAD-2	Severity of anxious symptoms in terms of frequency at the time of visit	− 0.05	0.0028	0.28
	CGI-S	Global severity of the overall presentation assessed by the clinician at the time of visit	− 0.05	0.0023	0.34
	CGI-I	Global improvement of the overall presentation assessed by the clinician	− 0.14	0.020	0.01
Assessment scores at 90 days^a^	ASSET	Severity of ADHD symptoms in terms of daily life functioning impact	− 0.03	0.00063	0.62
	PHQ-9	Severity of depressive symptoms in terms of frequency	− 0.00	0.000004	0.96
	GAD-2	Severity of anxious symptoms in terms of frequency	0.04	0.0013	0.47
	CGI-S	Global severity of the overall presentation assessed by the clinician	− 0.04	0.0014	0.45
	CGI-I	Global improvement of the overall presentation assessed by the clinician	− 0.10	0.0096	0.06
Treatment response (Change in clinical assessments from baseline to 90 days^a)^	Change in ASSET	Change of severity of ADHD symptoms in terms of daily life functioning impact	0.09	0.0074	0.08
	Change in PHQ-9	Change of severity of depressive symptoms	− 0.03	0.0011	0.50
	Change in GAD-2	Change of severity of anxiety symptoms	− 0.09	0.0083	0.06
	Change in CGI-S	Change of global severity of the overall presentation assessed by the clinician	− 0.05	0.00086	0.34

ASSET, ADHD Symptom and Side Effect Tracking; CGI-S, Clinical Global Impressions-Severity; CGI-I, Clinical Global Impressions-Improvement; ER, extended release; GAD-2, Generalized Anxiety Disorder 2-Item; PHQ-9, Patient Health Questionnaire-9

^a^ The visit falling closest to 90 days after ER stimulant was prescribed

**Table 5:** Binomial regressions demonstrating the relationship between each categorical variable and the addition of IR medication at 90 days

Variable Group	Variable	Wald statistic (χ^2^)	Nagelkerke *R*^2^	*p*
ER Prescription	Number of ERs previously attempted	1.40	0.011	0.24
	ER Prescription category (Dyanavel XR, AMP ER Stimulants, or MPH ER Stimulants)	2.40	0.017	0.12
	**ER Prescription category (pooled; Dyanavel XR or all other ER stimulants)**	**4.32**	**0.039**	**0.04**
Side effects at baseline^a^	Insomnia	0.06	0.000	0.81
	Generalized Pain	0.06	0.000	0.80
	Dry mouth	0.54	0.004	0.46
	Poor Appetite	2.07	0.016	0.15
	Food Binges	0.39	0.003	0.53
	Tics	0.00	0.000	0.96
	Anger	0.06	0.000	0.81
	Suspiciousness	0.63	0.005	0.43
	Restless Legs	0.28	0.002	0.60
	End of Dose Crash^b^	2.12	0.005	0.15
	Return of Symptoms as Medication Wears Off	3.55	0.057	0.06
Side effects at 90 days^a, c^	Insomnia	0.12	0.001	0.73
	Generalized Pain	0.33	0.002	0.57
	Dry mouth	2.5	0.020	0.12
	Poor Appetite	0.24	0.002	0.63
	Food Binges	0.05	0.000	0.83
	Tics	0.09	0.001	0.76
	Anger	2.33	0.018	0.13
	Suspiciousness	0.24	0.002	0.60
	Restless Legs	0.67	0.005	0.41
	End of Dose Crash^b^	2.22	0.015	0.14
	Return of Symptoms as Medication Wears Off	2.90	0.046	0.09

ER, extended release

^a^ Patient-rated Likert scales of how often the side effect was experienced in the past two weeks

^b^ Answered only if on ADHD medications

^c^ The visit falling closest to 90 days after ER stimulant was prescribed

### Backward elimination stepwise regression

The backward elimination stepwise regression started with 11 side effects reported at baseline (generalized pain, insomnia, fatigue, dry mouth, poor appetite, food binges, tics, anger, suspiciousness, restless legs, and end of dose crash) determined to be potential predictors of supplemental IR use at 90 days, as inadequate symptom management and side effects are key reasons for augmenting treatment in adults [36]. The initial regression was not significant (*F*_11,376_ = 0.690, *p* = .748). After the backward elimination procedure, the model did not reach significance with any predictor variable.

In another stepwise regression of the same 11 side effects reported at 90 days, the initial model was not significant (*F*_11,376_ = 0.841, *p* = .599). After backward elimination, the model trended to significantly predict IR supplementation at 90 days (*F*_13,387_ = 2.55, *p* = .055) when including the predictor variables dry mouth (*t* = -1.61, *p* = .11), anger (*t* = -1.44, *p* = .15), and end of dose crash (*t* = 1.97, *p* = .050), with end of dose crash significantly predicting IR use at 90 days. Continuing the stepwise elimination resulted in a failure of any predictor to reach statistical significance (all *p* > .05).

The regression including the change in CGI-S, CGI-I, ASSET, GAD-2, and PHQ-9 scores from baseline to 90 days was significant (*F*_5,365_ = 3.07, *p* = .010), indicating that the change in at least one assessment score affected IR use at 90 days. Change in CGI-S (*t* = 0.59, *p* = .95), CGI-I (*t* = -1.17, *p* = .25), and PHQ-9 (*t* = 0.21 *p* = .83) were not significant predictors of IR supplementation at 90 days. Change in ASSET scores, indicating improved ADHD symptom presentation, and change in GAD-2 scores, indicating worsened anxiety, predicted greater IR use at 90 days (ASSET: *t* = 3.01, *p* = .003; GAD-2: *t* = -2.38, *p* = .018).

After identifying ASSET and GAD-2 measures as significant predictors of IR use at 90 days, the next analysis sought to determine if Dyanavel XR could refine the predictive model. With the three predictor variables of change in ASSET, change in GAD-2, and Dyanavel XR, the model still significantly predicted IR use at 90 days (*F*_3,404_ = 4.81, *p* = .003), and all variables were significant predictors (ASSET change: *t* = 2.377, *p* = .018; GAD-2 change: *t* = -2.543, *p* = .011; Dyanavel XR: *t* = -2.112, *p* = .035). This demonstrates that together, negative change in ADHD symptoms and positive change in anxiety, in addition to being on Dyanavel XR was associated with reduced IR use at 90 days.

### Path analysis

**Fig. 1:**
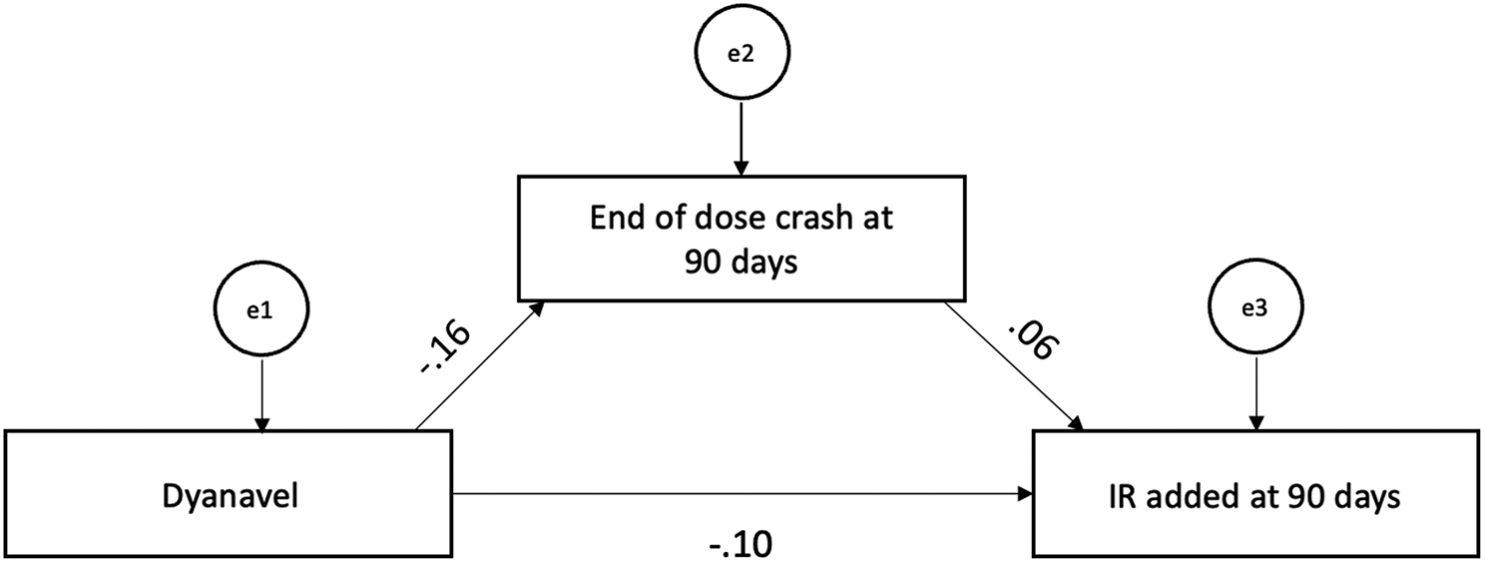
Path model showing the effects of Dyanavel and end of dose crash at 90 days on the addition of an IR stimulant at 90 days. The 90-day time point indicates the visit falling closest to 90 days after the ER stimulant was prescribed

Structural equation models and path analyses were planned to determine the relationships between significant variables. However, the results did not yield sufficient independently significant variables to attempt an adequately-powered analysis. In lieu of a complete analysis, the results prompted an exploratory path analysis to determine whether the relationship between Dyanavel XR and reduced IR supplementation at 90 days was explained in part by the relationship between the end of dose crash and IR supplementation. The path analysis shows that Dyanavel XR independently reduced the occurrence of end of dose crash and reduced IR supplementation. However, end of dose crash was not significantly associated with IR supplementation, demonstrating that the effect of Dyanavel XR on IR supplementation is not explained by its tendency to mitigate end of dose crashes (Table 6; Fig. 1).

**Table 6:** Path analysis showing relationships between Dyanavel, end of dose crash, and IR supplementation at 90 days

Regression weights	Estimate	SE	CR	*p*
End of dose crash^a^ ← Dyanavel XR	− 0.35	0.11	-3.30	< 0.001
IR supplementation ← End of dose crash^a^	0.01	0.01	1.22	0.22
IR supplementation^a^ ← Dyanavel XR	− 0.05	0.02	-2.03	0.04

CR, critical ratio; IR, immediate release; SE, standard error; XR, extended release

^a^ At the time of visit falling closest to 90 days after ER stimulant was prescribed

## Discussion

With the tremendous heterogeneity in the patient population with ADHD, different treatments may interact with individual patient characteristics and modify the effect of therapies on patient outcomes [11]. The present analyses aimed to determine whether a specific ER amphetamine stimulant, Dyanavel XR, was uniquely associated with the likelihood of patients supplementing ER treatment with an IR medication. The results show that over 90 days of treatment, compared to other ER amphetamines and ER methylphenidates, patients who were prescribed Dyanavel XR were less likely to supplement with IR formulations. Importantly, the association between Dyanavel XR and reduced IR supplementation was not explained by any other baseline patient variable measured here, but it was related to their change in ADHD and anxiety symptoms over 90 days.

The analyses also showed that patients who used IR medications at baseline were more likely to supplement with an IR medication at 90 days, regardless of which ER medication they were prescribed. Dyanavel XR was distinct from the other ER stimulants because it maintained an association with reduced IR supplementation even when controlling for patients’ use of IR medications at baseline. Previous studies have shown that patients taking ER stimulants have greater treatment adherence and are less likely to switch to or augment with a medication of a different release method [3–5, 37]. In a retrospective claims database analysis from 2010, patients taking ER amphetamines (Adderall XR, Dexedrine Spansules, or Vyvanse) had better treatment adherence and persistence than those taking ER methylphenidates (Concerta, Daytrana, or Focalin XR), but were equally likely to augment with IR medications [3]. This is consistent with the present results showing that only Dyanavel XR reduced the tendency to supplement with an IR medication. These results distinguish Dyanavel XR from other ER amphetamines in its potential to be used as monotherapy.

After confirming that Dyanavel XR reduced IR supplementation at 90 days, subsequent analyses investigated whether patient variables could better explain this association. The analyses did not reveal an impact of side effects reported at baseline on IR supplementation. Of the side effects reported at the 90-day visit, there was a trend to predict IR supplementation when the regression model was reduced to include only dry mouth, anger, and end of dose crash. In this model, the only significant predictor of IR use at 90 days was the frequency of experiencing an end of dose crash. Certainly, patients value medications with a longer duration of effect, shorter speed of onset, and reduced side effects [38]. Although reducing the risk of an end of dose crash, or rebound effect after medication wears off, is less important to patients than reducing headaches, insomnia, and mood changes, it is still considered an important factor [39]. Additionally, dose augmentation strategies are typically implemented when patients desire symptom management for longer than the typical 10–12 h achievable with ER medications and want to avoid rebound effects [18, 39, 40]. The results here are consistent with the literature showing that some side effects, especially rebound after a dose wears off, would lead patients to augment their daily treatment [36]. However, interpretation of this regression model is limited because the relationship was not strong and was driven by the effect of end of dose crash.

Patients’ response to treatment regarding their anxiety and ADHD symptoms predicted their likelihood of supplementing with an IR medication. The present analysis found that patients who were taking Dyanavel XR whose anxiety improved and ADHD symptomatology worsened over 90 days were less likely to supplement with IR medication. Given that anxiety is highly comorbid with ADHD, it is important to consider the effects one treatment may have on the presentation of both disorders [5, 38, 39, 41]. Some patients experience anxiety as a side effect of ADHD medications, and anxiety is one of the most common complications causing patients to discontinue their treatment [16, 36, 42]. The combination of long-term changes in anxiety and ADHD, along with being on Dyanavel XR, reduces the tendency to supplement with IR medication, which supports the impact of Dyanavel XR on maintaining monotherapy.

The present results reflect the heterogeneous nature of ADHD in the general population and could thus be confounded by variability due to genetic and environmental factors that were not measured in this study. Differences in which symptoms manifest and persist, as well as their severity, have been linked to prenatal and postnatal experiences related to maternal health, stressors during pregnancy, and psychosocial childhood adversity [11]. Exposure to harmful chemicals, toxins, and poor nutrition may also contribute to dysregulated neurobehavioral systems and give rise to ADHD-related symptoms [15]. As such, the etiology of ADHD is complex and relies on an interaction between inherited genetic traits, divergent neurobiology, and environmental risk factors that could not be fully captured with the study design presented here.

The clinical manifestation of ADHD may also vary over time within the same individual, as previous research has noted differences in which symptoms are likely to characterize the disorder depending on age [43]. ADHD is traditionally thought of as a childhood disorder, but diagnoses amongst adolescents and young adults have become more common, in part due to the recognition that symptoms can fluctuate across development [7, 44]. For example, restlessness, aggression, and disruptive behaviors are more common in young children, whereas inattention tends to persist as people get older [45, 46]. Additionally, age has been linked to treatment patterns, including initiation, switching, and discontinuation [47]. The present results showed that age at the start of the study was not significantly associated with IR supplementation at 90 days. Given the wide range of ages included from 18 to 81 years old, the age at which symptoms first appeared and age at first treatment initiation could have an effect, but the de-identified dataset precluded access to such historical data. Therefore, a direct investigation of how age at diagnosis, age at treatment initiation, and other demographic and environmental risk factors affect treatment responses would be a logical step for future research.

Several additional limitations should be considered in the interpretation of these results. As an observational study, the patient population was sampled to achieve balanced groups of current ER medications, but could not account for the heterogeneity in patients’ medication history. The patient population also lacked diversity, with most patients being white and having obtained at least an undergraduate degree, limiting our external validity. Additionally, relatively few patients in this sample added an IR medication at the 90-day visit, limiting analytical power to detect smaller effect sizes of patient-level variables that could be influencing IR supplementation. Future studies would benefit from the inclusion of a control group of patients who did not receive an ER medication. Because this was a non-experimental study using observational data from a given timeframe, there was a paucity of data available for individuals who were not treated medically.

Importantly, there was no significant difference in prescriber tendencies between the patients who did and did not add an IR medication, with both groups seeing clinicians who prescribed IR medications approximately one-fourth of the time. This rules out the potential that clinician biases were driving the results. However, longer-term follow-up would clarify whether Dyanavel XR prevents IR supplementation or delays it. Finally, these analyses relied on subjective psychometric tests for depression, anxiety, and ADHD, ratings of side effects, and clinician measures of improvement. Although these rating scales have been well-validated, psychiatry is always aiming to improve the reliability and validity of such measures [22, 30, 32]. 

ER stimulants are a first-line option for adults with ADHD because they lead to better treatment adherence and reduce the risk of misuse compared to IR formulations [41, 48, 49]. Still, some clinicians advise patients to supplement with an IR medication later in the day to ensure symptoms can be managed [7, 38, 48]. For adults who have responsibilities throughout the day, effective ER medications that can be reliable as monotherapy are preferred. Dyanavel XR utilizes a unique mechanism of sustained release, which resulted in an efficacy duration of up to 13 h in the double-blind clinical trials in children and adults [18, 50]. While direct comparisons are not possible without head-to-head clinical trials, the statistical analyses presented here using retrospective patient data support the benefit of Dyanavel XR in reducing the need to supplement with an IR medication, regardless of IR supplementation at baseline. Despite its limitations, this study contributes to the growing literature demonstrating the value of precision medicine in ADHD treatment. Although treating ADHD is complicated by the wide range of symptoms and responses shown in patients, predictive analyses, such as those shown here, can be translated to clinical care. Additional real-world investigations should be conducted to determine whether individual variables can predict treatment efficacy to promote data-driven individualized treatment plans for ADHD.

## Conclusions

Adults with ADHD desire consistent and extended symptom management without the need for multiple, supplementary medications. The current research shows that Dyanavel XR is uniquely associated with reductions in the tendency to supplement daily ER treatment with IR medications. Clinicians may consider these results when making treatment decisions with their adult patients with ADHD.

## Data Availability

The datasets used in the current study are available from the corresponding author upon reasonable request.

## Abbreviations

ADHD: Attention deficit hyperactivity disorder
ASSET: ADHD Symptom and Side Effect Tracking
CGI-I: Clinical Global Improvement-Improvement
CGI-S: Clinical Global Improvement-Severity
ER: Extended release
GAD-2: Generalized Anxiety Disorder-2
IR: Immediate release
PHQ-9: Patient Health Questionnaire-9
RCBM: Rochester Center for Behavioral Medicine
XR: Extended release
